# COVID-19 as a chance for hybrid teaching concepts

**DOI:** 10.3205/zma001408

**Published:** 2021-01-28

**Authors:** Yaël Weissmann, Mirdita Useini, Jörg Goldhahn

**Affiliations:** 1 ETH Zurich, Zurich, Switzerland

**Keywords:** hybrid teaching, teamwork, medical education, COVID-19

## Abstract

COVID-19 has turned the 2020 spring semester upside down. Three days before the start of the block week of the “Teamwork” module, the Federal Office of Public Health (FOPH) announced the ban on live interaction, which made it impossible to conduct five days of practice in the simulation centre of the University Hospital of Zurich. But how can the teaching of all the learning objectives necessary for medical training be guaranteed during an exceptional situation with constantly changing conditions? In the case of the BSc Human Medicine at ETH Zurich (ETHZ), the answer is: Hybrid teaching.

The field report “COVID-19 as a chance for hybrid teaching concepts” outlines how ETHZ switched to hybrid teaching within a very short time and how hospital placements were combined with video conferences. The qualitative surveys conducted at the end of the semester and the weekly quantitative surveys of students from March to June indicate the importance of personal exchange despite the ban on contact and that interactivity is possible even without physical proximity. An example from the autumn semester will also be used to show which aspects have proved to be successful and can therefore be retained.

## Zusammenfassung

COVID-19 hat das Frühlingssemester 2020 komplett auf den Kopf gestellt. Drei Tage vor Beginn der Blockwoche des Moduls „Teamwork“ gab das Bundesamt für Gesundheit (BAG) das Verbot von Live-Interaktion bekannt, was die Durchführung von fünf Übungstagen im Simulationszentrum des Universitätsspitals Zürich verunmöglichte. Doch wie kann die Vermittlung aller für die Medizinausbildung nötigen Lernziele während einer Ausnahmesituation mit stetig wechselnden Bedingungen garantiert werden? Im Falle des BSc Humanmedizin an der ETH Zürich (ETHZ) lautet die Antwort: hybrid.

Der Erfahrungsbericht „COVID-19 als Chance für hybride Lehrkonzepte“ skizziert, wie an der ETHZ in kürzester Zeit die Umstellung auf hybriden Unterricht erfolgte und Spitaleinsätze mit Videokonferenzen gepaart wurden. Die qualitativen Befragungen Ende des Semesters sowie die von März bis Juni wöchentlichen quantitativen Erhebungen der Studierenden deuten auf die Wichtigkeit von persönlichem Austausch trotz Kontaktverbot hin und dass Interaktivität auch ohne physische Nähe möglich ist. Anhand eines Beispiels aus dem Herbstsemester wird ausserdem aufgezeigt, welche Aspekte sich bewährt haben und beibehalten werden können.

## 1. Introduction

Three days before the start of the block week of the “Teamwork” module, the Federal Office of Public Health announced the ban on live interaction, which made it impossible to conduct five days of practice in the simulation centre of the University Hospital of Zurich. The teamwork module would have addressed the role of a collaborator (“As collaborators, physicians are teamplayers who effectively work together in interdisciplinary and interprofessional partnerships in order to provide optimum patient care, education, and/or research” [1]) in the CanMed model (Profiles GO 3) in simulations of varying complexity and demonstrated that interdisciplinary cooperation and good communication are fundamental for successful treatment. A further aim of the module, based on the Cognitive Apprenticeship Method, was to be able to put one’s own thoughts behind the actions into the context of daily work [2]. A simultaneous request from the cantonal medical officers allowed the students to provide on-site support during the exceptional situation in the hospitals and therefore to test their ability to work in a team and deal with delicate stress situations in real life.

## 2. Monitoring of COVID-19 placements

From March to June 2020, 69 students in the 6t^h^ semester of the Bachelor's degree programme in Human Medicine at ETHZ were working in different hospitals to do their part in the fight against the novel coronavirus. Their tasks ranged from admission control, anamnesis in the temporary triage and assistance in the laboratory to support in the intensive care unit. 

In order to categorise the strongly varying personal insights of the students and thereby identify general developments and patterns, weekly lessons learned were collected via e-mail. In the first few weeks, many students described how they were able to integrate the communication skills they had learned in theory into everyday life in the clinic and how they recognised the importance of empathy and adaptability. Over time, the focus gradually shifted to their own clinical skills and everyday hospital life, especially the overtime that had to be worked and the willingness to compromise.

During the placements, some students found themselves in situations they were not prepared for. In order to be able to classify such experiences correctly, they had the opportunity to report on their experiences at the beginning of the daily 90-minute Zoom coaching sessions and to reflect on them in a protected setting with their fellow students and lecturers. The remaining time was used to deal intensively with different topics: Speak Up in critical situations, briefings & debriefings, time-outs and boards & colloquia. As a preparation, the students received a separate work assignment for each topic and had to inform themselves beforehand using specialised texts and videos, which ensured the common thread and the professional classification. The lecturers were also available for meetings via Zoom after the end of the module. 

Qualitative surveys of 12 students have shown that these meetings were highly appreciated. The students benefited from each other's experiences and were able to better classify and process significant or frustrating moments. Since the curriculum is based on the principle of contextual learning, this course unit fitted well into the overall concept of the programme [3]. With a little more lead time, a more structured approach and more specific tasks to achieve the learning objectives, this form of practical learning could be used even more effectively. The comparison of expectations and experience can lead to a better classification of teamwork in everyday life and thereby to a better understanding of the role of the collaborator in the CanMed model. The reflection should therefore be further developed in the next implementation.

Since ETHZ had already been using the video conferencing service Zoom for transmissions before COVID-19 and ETHZ was able to increase the number of licences in a very short time without difficulty, its use for teaching purposes was obvious. Especially the breakout rooms, in which participants can be divided into small groups for discussions or meetings for a certain period of time, make it possible to do justice to the current conditions and still maintain a high level of interaction. The experience of ETHZ corresponds with the experiences of other institutions [4], [5].

## 3. Simulation vs. practice: what remains?

The hospital placements have shown that medical training does not work without real situations. Intensive interpersonal interactions with patients, relatives and one’s own team cannot be simulated virtually in all their complexity, nor can you experience your own stress limits. The question of when practice and when simulation is most appropriate and how the two methods can be combined best will accompany us for the time being. This can clearly be seen in the current example of the introductory week of the module "Fundamentals of Medicine and the Human Body". Instead of five days in the clinic, this year’s module was switched to hybrid teaching. Students are taught the most important theoretical aspects via Zoom, before they then move around the hospital in small groups for half a day. On the last day, a joint virtual debriefing takes place for all of them. When planning this week, we were able to benefit greatly from the experience gained in March.

Through COVID-19, we are forced to rethink the teaching methods, such as the combination of distance learning via video technology with small on-site group lessons – a seemingly effective learning combination. Now it is time to build on the experiences and, through close monitoring of teaching research, to switch to hybrid teaching concepts in the long term.

## Competing interests

The authors declare that they have no competing interests. 
